# Influence of malocclusion on OHRQoL in adolescents in initial orthodontic treatment phase

**DOI:** 10.1007/s00784-024-05689-0

**Published:** 2024-04-30

**Authors:** Linda Schwarz, Victor Ossmann, Valentin Ritschl, Tanja Stamm, Erwin Jonke, Katrin Bekes

**Affiliations:** 1 Department of Orthodontics, Medical University Vienna, University Clinic of Dentistry, Sensengasse 2a, Vienna, 1090 Austria; 2 Department of Paediatric Dentistry, Medical University Vienna, University Clinic of Dentistry, Sensengasse 2a, Vienna, 1090 Austria; 3 Center for Medical Data Science, Institute for Outcomes Research, Medical University of Vienna, Ludwig Boltzmann Institute for Arthritis and Rehabilitation, Spitalgasse 23, Vienna, 1090 Austria

**Keywords:** Oral health-related quality of life (OHRQoL), Child perceptions questionnaire (CPQ), Orthodontic treatment

## Abstract

**Objectives:**

Besides correcting malocclusions, another main objective of orthodontic treatment is to improve patients’ oral health-related quality of life (OHRQoL). This study aimed to assess changes in OHRQoL of children within the first six months of orthodontic therapy with fixed orthodontic appliances.

**Methods:**

85 patients aged 11 to 14 years requiring fixed orthodontic appliance therapy were included. The children completed the German version of the Child Perceptions Questionnaire (CPQ-G-11-14) before (T0), 1 month (T1) and 6 months (T2) after the start of orthodontic treatment. The type of malocclusion was categorized according to the Index of Orthodontic Treatment Need (IOTN).

**Results:**

The initial type of malocclusion affected the children’s OHRQoL, whereas gender and age did not. The IOTN dental health component (DHC) had a significant impact on the CPQ score (median CPQ of 15.00 for the group DHC 4 vs. 22.50 for DHC 5, *p* = 0.032). The onset of orthodontic treatment initially affected the CPQ domains “Oral symptoms” and “Functional limitations, with a change versus baseline of 2.00 (p = 0.001), but improved again after 6 months. Regression analysis demonstrated that children with an IOTN DHC 5 malocclusion experienced a greater impact on their ORHQoL, as indicated by a CPQ score 7.35 points higher than that of children with an IOTN DHC 4 malocclusion (p = 0.015).

**Conclusions:**

At the beginning of orthodontic treatment, the OHRQoL slightly worsens, probably due to the discomfort and appearance of the appliances. However, 6 months after the start of orthodontic treatment, OHRQoL improved again in patients with severe malocclusion (IOTN 4 and 5), and approached baseline values.

**Clinical relevance:**

The results help the clinician to better understand specific aspects of oral health that may be affected by different malocclusions, thereby improving the child’s satisfaction and overall quality of life.

## Introduction

Quality of Life (QoL) has been widely validated as a parameter for patient-centred oral health care assessment [1]. According to the WHO, oral health is based on clinical variables, such as caries indices or periodontal parameters, and general well-being and the ability to eat and speak [2]. Assessment of Oral Health-Related Quality of Life (OHRQoL) reflects the interaction of a person’s oral health condition with social and contextual factors [3], therefore representing a person’s subjective perspective. Thus, it is possible to understand the patient’s psychosocial resources and oral health behaviours. To assess the influence of oral health on patients’ quality of life, several measures of OHRQoL have been developed [4–6]. They allow patients to rate their OHRQoL and to which extent oral disorders affect their functional and psychosocial well-being [7].

A malocclusion is a misalignment or irregularity of the teeth or jaw and can be treated with either fixed or removable orthodontic appliances. Severe malocclusions, especially in the aesthetic zone, do not only lead to functional limitations [8], but also negatively impact the social and emotional well-being of children and adolescents [8–10]. This aligns with the primary factors that motivate patients to undergo orthodontic treatment: a desire for improved aesthetics, enhanced functionality, and relief from pain [11]. As a result, orthodontic treatment not only aims to enhance patients’ oral health [12] but is also initiated with the expectation that it will lead to improved dental aesthetics and emotional well-being. Consequently, it aims to enhance the individual’s overall oral health-related quality of life (OHRQoL) [12–14].

Currently, the inclusion of patient-reported measures in orthodontic research is increasing; these include pain during treatment, expectations of treatment and quality of life items (impact of malocclusion, acceptability of treatment, anxiety and occlusion [15]. An orthodontic appliance may affect OHRQoL through functional limitations, pain, discomfort and emotional and social well-being [16, 17], even after the initial separation of molars [18]. The greatest impact on quality of life is in the first six months of treatment [17], particularly in the first week after bonding [16]. Therefore, evaluating these aspects is important for understanding patients’ perceptions of orthodontic treatment and can help to identify treatment needs.

Due to the limited number of longitudinal studies available, the evidence regarding the influence of orthodontic treatment on OHRQoL is considered to be of moderate quality [13]. Longitudinal changes in OHRQoL have been reported but their responsiveness has not been sufficiently investigated [19, 20]. The inclusion of clinical data has been recommended to enhance interpretability [20]. Thus, the study´s objective was to assess changes in adolescents’ OHRQoL during the initial treatment phase, specifically at 4 weeks and 6 months after bonding of the fixed appliance. It is the first prospective longitudinal trial assessing OHRQoL of Austrian adolescents undergoing orthodontic treatment. The specific aim of this study was to test for the influence of malocclusion type on OHRQoL changes in the course of orthodontic treatment to provide subgroup scores for further interpretation and to validate the changes by use of effect size determination [21] .

## Materials and methods

### Subjects and setting

This prospective study was conducted at the Department of Orthodontics at the University Clinic of Dentistry in Vienna, Medical University of Vienna, Austria. The study protocols received approval from the ethics committee of the local University Review Board (Medical University of Vienna, #1501–2016).

Over a period of 32 months, a consecutive sample of 85 children aged 11 to 14 years, who showed a significant need for orthodontic treatment involving fixed appliances were recruited. All patients in this study received treatment with buccal metal brackets (Empower, American Orthodontics, Wisconsin, USA). To be eligible for participation in the study, children had to meet the following inclusion criteria: 10–14 years of age, entitlement to orthodontic treatment covered by public health insurance, absence of craniofacial anomalies and no history of prior orthodontic treatment. Enrolment in this study was entirely voluntary. Comprehensive information leaflets explaining the study’s objectives were provided to parents and children. Children and their parents or legal guardians were required to sign an informed consent form at enrolment. If a child expressed dissent, it superseded the parental consent.

The sample size calculation was derived from the findings of two prior studies [16, 22], which recommended a sample size of 80 to achieve a statistical power of 80% at a significance level of *p* ≤ 0.05 for detecting statistical differences. To account for an anticipated dropout rate of 10%, a sample size of 85 was selected.

### Data collection

The children underwent clinical examinations conducted by two examiners calibrated according to the WHO basic methods criteria [23]. The DMFT index (sum of decayed, missing, and filled teeth in the permanent dentition) and its components were used to assess the caries status. For the objective evaluation of orthodontic treatment need, the dental health component (DHC) and the aesthetic component (AC) of the Index of Orthodontic Treatment Need (IOTN) [24] were used. The DHC specifically focuses on occlusal traits and ranges from “no need” of treatment (IOTN DHC 1) to “great need” (IOTN DHC 5). To assess IOTN DHC, digital casts were acquired either by scanning conventional plaster models or by using digital intra-oral scanning methods.

The Software OnyxCeph^3^ (Image Instruments GmbH, Chemnitz, Germany) was utilized to analyse the digital models. To assess IOTN AC, a dental attractiveness scale consisting of 10 intraoral photographs with decreasing attractiveness was used. Evaluations were conducted by matching the patient’s dental attractiveness of the patient to one of the provided photographs.

The validated German version of the Child Perceptions Questionnaire (CPQ) for 11-to 14-year-old children (CPQ-G11-14) was used to assess children’s OHRQoL [25]. The CPQ-G11-14 comprises four domains and a total of 35 questions: five questions related to oral symptoms, ten questions on functional limitations, eight questions on emotional well-being, and 12 questions on social well-being. Domain scores and an overall CPQ11–14 score were generated by summing the response codes for the questionnaire items. Higher scores indicate a poorer OHRQoL status. The summary score for this instrument ranges from 0 to 140. A score of zero indicates the absence of any problems, higher CPQ scores reflect a more impaired OHRQoL. Additionally, the CPQ-11-14 includes two questions asking the child to provide a global rating of their oral health and overall well-being, with a five-point response format (excellent, very good, good, moderate, poor).

Children were asked to complete the questionnaire at various time points: before treatment (T0), 1 month (T1), and 6 months (T2) after the insertion of the fixed orthodontic appliance. During the initial appointment, participants were also asked three additional questions about their previous experiences with orofacial pain, history of dental trauma, and participation in a preventive dental care program.

### Statistical analysis

The data were collected and analysed using the software “R” 4.2.2 (R Foundation for Statistical Computing, Vienna, Austria). The Wilcoxon signed-rank test was used to compare baseline and follow-up scores and assess the statistical significance of the changes. The effect size was determined by dividing the mean of change score by the standard deviation of the baseline score, serving as an approximation of the MID (Minimal Important Difference) [21]. An effect size of < 0.2 indicated a trivial magnitude of change, while an effect size in the range of 0.2 to 0.7 represented a moderate change, and an effect size exceeding 0.7 signified a large change. Linear regression analysis was employed to determine the impact of possible confounding factors on the change in CPQ scores during the initial orthodontic treatment phase, specifically at the 6-month mark following the insertion of the orthodontic appliance.

## Results

In total, 85 children aged 11–14 years were recruited. No children were excluded due to missing items surpassing the permissible threshold in the CPQ-11-14 questionnaire. Thus, 85 children were included in our analysis, with a mean age of 12.1 ± 1.0 years. 48.2% of the children included were female (Table 1).

**Table 1:** General characteristics of participants at baseline

Characteristics	*N* (%)
Gender	*N* = 85
Male	44 (51.8%)
Female	41 (48.2%)
Age group	
10 years	3 (3.5%)
11 years	20 (23.5%)
12 years	34 (40.0%)
13 years	20 (23.5%)
14 years	8 (9.5%)
DMFT Score, median[IQR]	1.00 [0.00, 4.00]
dmft Score, median[IQR]	0.00 [0.00, 0.00]
Treatment need (AC)	
1	10 (11.8%)
2	17 (20.0%)
3	20 (23.5%)
4	18 (21.2%)
5	3 (3.5%)
6	3 (3.5%)
7	2 (2.4%)
8	9 (10.6%)
9	3 (3.5%)
Treatment need (DHC)	
4.h	8 (9.4%)
4.a	21 (24.7%)
4.c	2 (2.4%)
4.l,	2 (2.4%)
4.d	22 (25.9%)
4.f	3 (3.5%)
4.t	1 (1.2%)
5.i	18 (21.2%)
5.h	2 (2.4%)
5.a	6 (7.1%)

### Baseline data

Female and male participants did not achieve statistically different CPQ scores (i.e. CPQ total, oral symptoms, functional limitations, social well-being and emotional well-being) (Table 2). At baseline, male adolescents had a median CPQ total score of 16.50 [9.00, 24.25] and female participants had a median score of 16.00 [8.00, 27.00]. Age did not significantly affect the CPQ scores, although there was a trend towards worse CPQ scores with increasing age: The baseline median CPQ score of 10-year-olds was 13.00 [10.00, 17.50] compared to a score of 21.50 [15.00, 27.50] for 14-year-olds. However, the children´s perceived OHRQoL worsened significantly along with the severity of the initial malocclusion (IOTN DHC): Children with IOTN DHC 4 achieved an overall median CPQ score of 15.00 [8.00, 20.50], whereas children with IOTN DHC 5 scored 22.50 [12.25, 32.25] (*p* = 0.032). The DMFT index did not affect the children´s OHRQoL (Table 2).

**Table 2:** CPQ scoring at baseline T0

		CPQ total	Oral symptoms	Functional limitations	Social well-being	Emotional well-being
All, mean (± SD)		17.65 (± 11.06)	4.66 (± 2.70)	4.02 (± 3.89)	3.99 (± 4.07)	4.98 (± 4.60)
Gender						
	Male, mean(± SD); median[IQR]	17.05 (± 10.48); 16.50 [9.00, 24.25]	4.57 (± 2.90); 4.50 [1.75, 6.25]	4.39 (± 3.60); 4.00 [2.00, 7.00]	3.82 (± 3.79); 3.00 [1.00, 5.25]	4.27 (± 3.93); 3.00 [1.75, 6.00]
	Female, mean(± SD); median[IQR]	18.29 (± 11.74); 16.00 [8.00, 27.00]	4.76 (± 2.49); 5.00 [3.00, 6.00]	3.63 (± 4.19); 3.00 [1.00, 5.00]	4.17 (± 4.39); 3.00 [2.00, 5.00]	5.73 (± 5.17); 5.00 [2.00, 9.00]
	*p*-value	0.771	0.884	0.183	0.733	0.217
Age						
	10 years, mean(± SD); median[IQR]	14.00 (± 7.55); 13.00 [10.00, 17.50]	3.33 (± 1.53); 3.00 [2.50, 4.00]	1.33 (± 1.15); 2.00 [1.00, 2.00]	4.00 (± 3.46); 2.00 [2.00, 5.00]	5.33 (± 5.03); 6.00 [3.00, 8.00]
	11 years, mean(± SD); median[IQR]	17.10 (± 9.35); 16.00 [10.75, 22.25]	4.55 (± 2.61); 4.00 [2.75, 6.00]	4.10 (± 4.27); 3.00 [1.00, 6.25]	4.20 (± 4.32); 2.50 [1.00, 5.25]	4.25 (± 3.78); 4.00 [1.00, 6.00]
	12 years, mean(± SD); median[IQR]	17.56 (± 11.57); 16.50 [8.00, 26.75]	4.79 (± 2.73); 5.00 [3.00, 6.00]	4.41 (± 4.26); 4.00 [2.00, 5.75]	3.82 (± 4.36); 3.00 [1.00, 5.00]	4.53 (± 4.25); 3.00 [1.25, 6.00]
	13 years, mean(± SD); median[IQR]	16.90 (± 11.97); 14.50 [5.00, 25.50]	4.20 (± 286); 4.00 [1.00, 6.25]	3.40 (± 2.76); 3.50 [1.00, 5.25]	4.00 (± 4.04); 3.00 [0.75, 6.50]	5.30 (± 5.17); 3.50 [1.75, 8.00]
	14 years, mean(± SD); median[IQR]	22.62 (± 12.52); 21.50 [15.00, 27.50]	6.00 (± 2.73); 5.50 [4.75, 7.25]	4.75 (± 4.50); 5.50 [0.00, 8.00]	4.12 (± 3.27); 3.00 [2.75, 4.00]	7.75 (± 6.23); 6.50 [4.50, 8.25]
	*p*-value	0.764	0.516	0.551	0.968	0.513
IOTN						
	DHC 4, mean(± SD); median[IQR]	15.54 (± 9.15); 15.00 [8.00, 20.50]	4.44 (± 2.51); 4.00 [2.50, 6.00]	3.51 (± 2.94); 3.00 [1.00, 6.00]	3.39 (± 3.54); 2.00 [1.50, 4.00]	4.20 (± 3.69); 3.00 [1.50, 6.00]
	DHC 5, mean(± SD); median[IQR]	22.42 (± 13.50); 22.50 [12.25, 32.25]	5.15 (± 3.08); 5.00 [3.00, 6.00]	5.19 (± 5.36); 3.50 [2.00, 6.75]	5.35 (± 4.89); 5.00 [1.00, 8.50]	6.73 (± 5.92); 6.00 [2.00, 10.00]
	*p*-value	0.032*	0.379	0.215	0.098	0.079
	AC 1–4, mean(± SD); median[IQR]	17.17(± 10.87); 16.00 [8.00, 26.00]	4.78 (± 2.86); 5.00 [3.00, 6.00]	3.98 (± 3.82); 3.00 [1.00, 6.00]	3.57 (± 3.57); 3.00 [1.00, 5.00]	4.83 (± 4.67); 4.00 [1.00, 7.00]
	AC 5–7, mean(± SD); median[IQR]	17.88 (± 10.05); 19.50 [10.50, 23.75]	4.12 (± 2.10); 4.50 [2.75, 6.00]	4.50 (± 5.48); 3.00 [1.00, 5.25]	4.75 (± 4.06); 3.50 [1.75, 8.25]	4.50 (± 4.78); 2.50 [1.50, 7.00]
	AC 8–10, mean(± SD); median[IQR]	20.08 (± 13.20); 18.00 [10.00, 26.25]	4.33 (± 2.19); 4.50 [2.75, 6.00]	3.92 (± 3.40); 2.00 [2.00, 6.25]	5.75 (± 6.08); 4.50 [2.00, 6.50]	6.08 (± 4.32); 5.50 [3.00, 7.75]
	*p*-value	0.804	0.867	0.979	0.415	0.470
DMFT						
	0, mean(± SD); median[IQR]	18.32 (± 12.21); 17.00 [7.50, 27.50]	4.61 (± 2.82); 5.00 [2.50, 6.00]	4.16 (± 2.95); 4.00 [1.50, 7.00]	4.48 (± 4.58); 3.00 [1.50, 6.00]	5.06 (± 5.27); 3.00 [1.00, 7.00]
	> 0, mean(± SD); median[IQR]	17.26 (± 10.44); 16.00 [9.00, 23.75]	4.69 (± 2.65); 4.50 [3.00, 6.00]	3.94 (± 4.37); 3.00 [1.00, 5.75]	3.70 (± 3.76); 3.00 [1.00, 5.00]	4.93 (± 4.22); 4.50 [2.00, 6.75]
	*p*-value	0.841	0.927	0.286	0.501	0.794

* Significantly different *p* < 0.05

### Changes of OHRQoL during orthodontic treatment

**Fig. 1:**
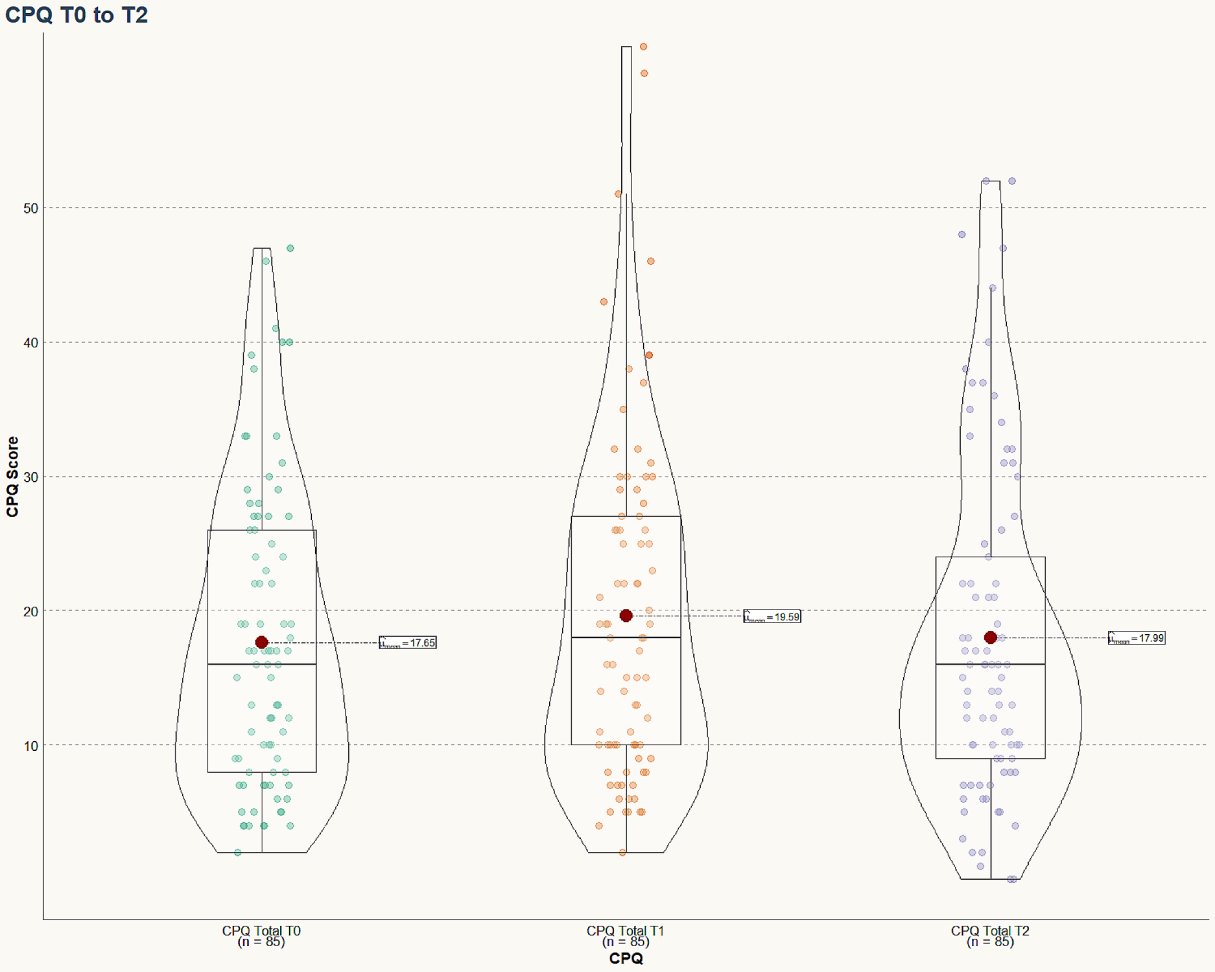
Violin plots of the total CPQ score before orthodontic treatment (T0), after 1 month of treatment (T1) and after 6 months of treatment (T2). The red dot marks the mean CPQ score, the box displays the interquartile range and the median

**Fig. 2:**
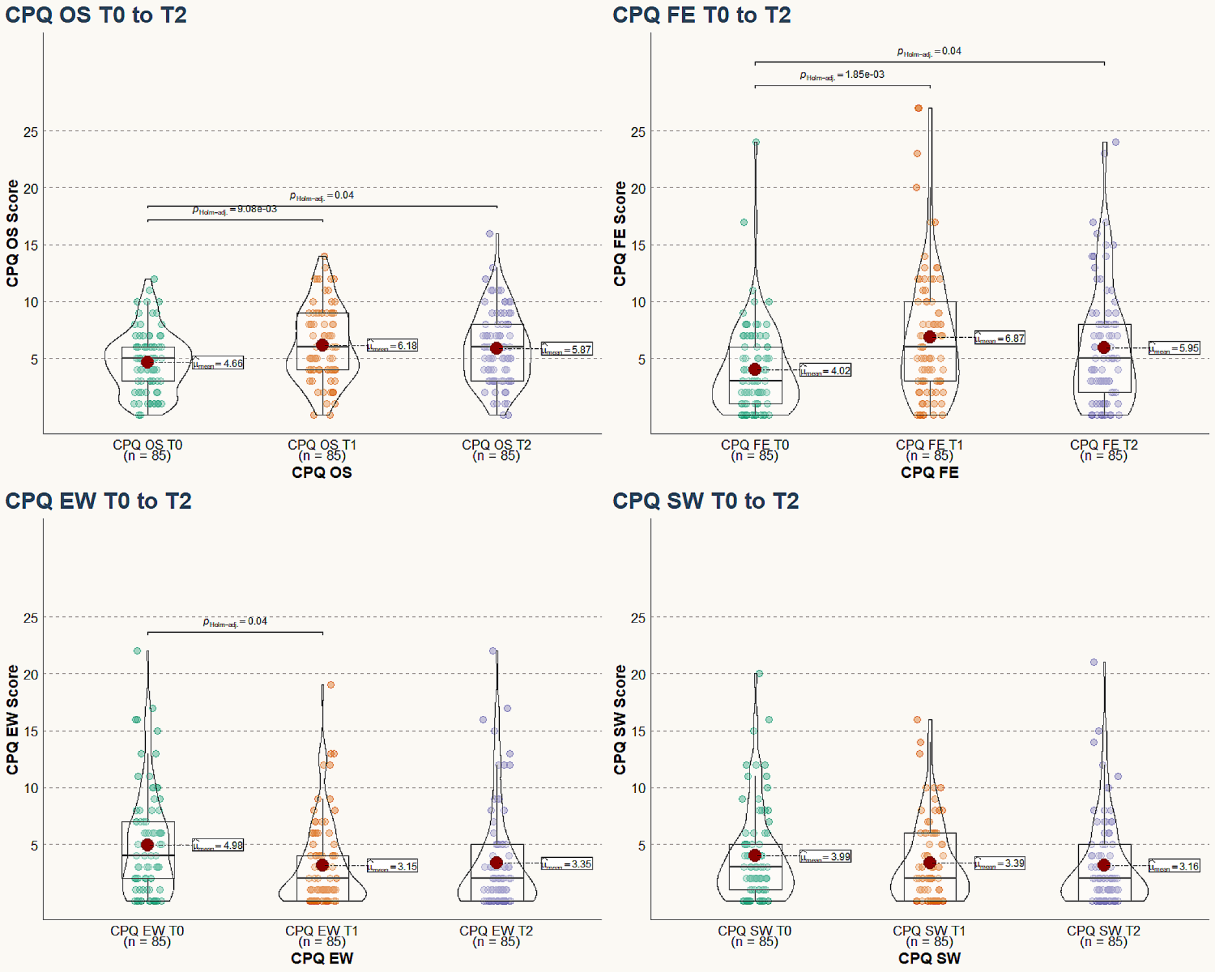
Violin plots of the four CPQ subdomains before orthodontic treatment (T0), after 1 month of treatment (T1) and after 6 months of treatment (T2). The red dot marks the mean CPQ score, the box displays the interquartile range and the median. Abbreviations: CPQ OS, oral symptoms; CPQ FE, functional limitations; CPQ EW, emotional well-being; CPQ SW, social well-being

Orthodontic treatment initially worsened OHRQoL, leading to an increase of the median total CPQ score from 16.00 [8.00, 26.00] to 18.00 [10.00, 27.00] after one month (Table 3; Fig. 1). However, this effect improved 5 months later: 1 month after start of treatment, the total CPQ increased by a median value of 2 points and 6 months after start of treatment, the median increase in CPQ was 0.00 points. 1 month after start of orthodontic treatment, both the domains “oral symptoms” and “functional limitations” worsened significantly by 2.00 points. In contrast, the “emotional well-being” domain improved significantly by 1.00 point (Table 3; Fig. 2).

**Table 3:** Changes in CPQ total scores and subdomains

	CPQ total	Oral symptoms	Functional limitations	Social well-being	Emotional well-being
Before treatment/ baseline (T0), mean(± SD); median[IQR]	17.65 (± 11.06); 16.00 [8.00, 26.00]	4.66 (± 2.70); 5.00 [3.00, 6.00]	4.02 (± 3.89); 3.00 [1.00, 6.00]	3.99 (± 4.07); 3.00 [1.00, 5.00]	4.89 (± 4.60); 4.00 [2.00, 7.00]
After 1 month (T1), mean(± SD); median[IQR]	19.59 (± 12.76): 18.00 [10.00, 27.00]	6.18 (± 3.22); 6.00 [4.00, 9.00]	6.87 (± 5.71); 6.00 [3.00, 10.00]	3.39 (± 3.53); 2.00 [0.00, 6.00]	3.15 (± 3.70); 2.00 [0.00, 4.00]
After 6 months (T2), mean(± SD); median[IQR]	17.99 (± 12.54); 16.00 [9.00, 24.00]	5.87 (± 3.18); 6.00 [3.00, 8.00]	5.95 (± 5.32); 5.00 [2.00, 8.00]	3.16 (± 3.97); 2.00 [0.00, 5.00]	3.35 (± 4.54); 2.00 [0.00, 5.00]
*p*-value	0.608	0.007*	0.001*	0.164	0.002*
Change versus baseline after 1 month, mean(± SD); median[IQR]; p-value	1.94 (± 11.68); 2.00 [-5.00, 10.00]; ns	1.52 (± 3.18); 2.00 [0.00, 3.00]; < 0.001*	2.85 (± 5.26); 2.00 [-1.00, 6.00]; 0.001*	-0.60 (± 3.83); 0.00 [-2.00, 1.00]; ns	-1.82 (± 3.58); -1.00 [-4.00, 0.00]; 0.002*
Change versus baseline after 6 months, mean(± SD); median[IQR]; p-value	0.34 (± 10.60); 0.00 [-6.00, 8.00]; ns	1.21 (± 3.25); 1.00 [-1.00, 3.00]; 0.008*	1.93 (± 4.53); 1.00 [-1.00, 4.00]; 0.054	-0.82 (± 4.03); 0.00 [-2.00, 1.00]; ns	-1.62 (± 4.16); -2.00 [-4.00, 0.00]; 0.002*

* Significantly different *p* < 0.05

The DHC 4 group reported an increase in the CPQ score with a median of 2.00 [-3.00, 9.50] points after 1 month and close to baseline values after 6 months (Table 4). Patients with DHC 5 malocclusion had the highest CPQ score at baseline (median 22.50), which increased by a median of 0.50 after one month and by 5.00 after six months. The DHC 4 and 5 groups had significantly different CPQ scores at baseline (median of 15.00 vs. 22.50; *p* = 0.032) and after six months of treatment (13.00 vs. 17.50; *p* = 0.035). Both the DHC 4 and DHC 5 group reported the highest OHRQoL after one month of treatment, with a median CPQ value of 16.00 and 23.50, respectively. No differences in the perception of changes in OHRQoL were reported between boys and girls (Table 4). At baseline, boys reported a median overall CPQ scoring of 16.50 and girls a CPQ of 16.00 (*p* = 0.77). After one month of treatment, the CPQ scorings rose to 18.50 in boys and 17.00 in girls (*p* = 0.735) and after 6 months, CPQ scorings approximated baseline values with 16.00 in boys and 15.00 in girls (*p* = 0.487).

Subgroup analysis of the most frequently observed IOTN DHC gradings (DHC 4a, 4d and 5i) revealed a significantly different perception of OHRQoL at baseline (Table 5). Children with DHC 5i grading (designated to patients with impeded eruption of teeth) had the lowest OHRQoL (median CPQ score of 24.00). Before treatment, there was a statistically significant difference between the DHC 4a, 4d and 5i groups (Table 5, *p* = 0.004). We conducted a post-hoc test, revealing significant differences between grades 4.a and 4.d compared to grade 5.i (*p* = 0.016 and *p* = 0.050, respectively), but not between grades 4.a and 4.d (*p* = 0.853). However, this difference was no longer significant after one month (*p* = 0.060) and six months of treatment (*p* = 0.104). The changes in CPQ scores during orthodontic treatment did not differ significantly between the DHC gradings (Table 5).

**Table 4:** Impact of orthodontic treatment need and sex on changes in CPQ score

	Before treatment (T0)	After 1 month (T1)	After 6 months (T2)	Change versus baseline after 1 month	Change versus baseline after 6 months
DHC 4 (*n* = 59), mean (± SD); median[IQR]	15.54 (± 9.15) 15.00 [8.00, 20.50]	17.86 (± 10.78) 16.00 [10.00, 25.00]	15.78 (± 10.87) 13.00 [8.00, 21.50]	2.32 (± 11.21) 2.00 [-3.00, 9.50]	0.24 (± 9.99) 0.00 [-5.00, 5.00]
DHC 5 (*n* = 26), mean (± SD); median[IQR]	22.42(± 13.50) 22.50 [12.25, 32.25]	23.50 (± 15.95) 23.50 [10.25, 33.75]	23.00 (± 14.70) 17.50 [12.00, 32.75]	1.08 (± 12.86) 0.50 [-7.75, 8.75]	0.58 (± 12.08) 5.00 [-8.75, 10.00]
*p*-value	0.032*	0.186	0.035*	0.333	0.616
Girls (*n* = 41), mean (± SD); median[IQR]	18.29 (± 11.74); 16.00 [8.00, 27.00]	19.56 (± 13.84) 17.00 [10.00, 28.00]	17.41 (± 13.33) 15.00 [7.00, 22.00]	1.27 (± 11.48) 2.00 [-4.00, 10.00]	-0.88 (± 11.89) -2.00 [-9.00, 5.00]
Boys (*n* = 44), mean (± SD); median[IQR]	17.05 (± 10.48) 16.50 [9.00, 24.25]	19.61 (± 11.81) 18.50 [10.00, 26.25]	18.52 (± 11.88) 16.00 [10.00, 25.25]	2.57 (± 11.95) 1.00 [-6.25, 9.25]	1.48 (± 9.23) 4.00 [-4.25, 8.00]
*p*-value	0.771	0.735	0.487	0.912	0.194

* Significantly different *p* < 0.05

**Table 5:** Impact of orthodontic treatment need on changes in CPQ score (mean (± standard deviation) and median[IQR]): Subgroup analysis

	DHC 4a (*n* = 21)	DHC 4d (*n* = 22)	DHC 5i (*n* = 18)	*p*-value
Before treatment/ baseline (T0), mean(± SD); median[IQR]	14.76 (± 10.33) 12.00 [7.00, 22.00]	16.77 (± 9.02) 15.50 [10.25, 21.25]	26.17 (± 12.79) 24.50 [16.25, 36.75]	0.008*
After 1 month (T1), mean(± SD); median[IQR]	15.24 (± 8.38) 15.00 [9.00, 22.00]	19.09 (± 9.85) 18.50 [10.00, 26.75]	26.33 (± 16.30) 24.00 [13.00, 37.25]	0.060
After 6 months (T2), mean(± SD); median[IQR]	14.71 (± 11.11) 12.00 [8.00, 18.00]	16.95 (± 11.02) 13.50 [10.25, 24.75]	24.50 (± 15.76) 19.50 [12.00, 32.75]	0.104
Change versus baseline after 1 month, mean(± SD); median[IQR]	0.48 (± 11.49) 2.00 [-4.00, 5.00]	2.32 (± 10.49) 4.00 [-3.25, 9.75]	0.17 (± 14.66) -1.50 [-8.00, 8.75]	0.530
Change versus baseline after 6 months, mean(± SD); median[IQR]	-0.05 (± 12.36) 2.00 [-4.00, 5.00]	0.18 (± 9.14) -2.00 [-5.00, 7.75]	-1.67 (± 13.00) -3.50 [-10.75, 8.75]	0.914

* Significantly different *p* < 0.05

**Table 6:** Effect Size of clinical meaningful change of CPQ and subdomains

	After 1 month (T0-T1)	After 6 months (T0-T2)
Overall CPQ, Cohen’s d [95%CI]	-0.2 [-0.5; 0.1]	0.0 [-0.3; 0.3]
Oral symptoms, Cohen’s d [95%CI]	-0.5 [-0.8; -0.2]	-0.4 [-0.7; -0.1]
Functional limitations, Cohen’s d [95%CI]	-0.6 [-0.9; 0.3]	-0.4 [-0.7; -0.1]
Social well-being, Cohen’s d [95%CI]	0.2 [-0.1; 0.5]	0.2 [-0.1; 0.5]
Emotional well-being, Cohen’s d [95%CI]	0.4 [0.1; 0.7]	0.3 [0.1; 0.7]

Small change < 0.2, moderate change 0.2–0.7 and large change > 0.7

Estimating a clinically meaningful change in CPQ using the effect size resulted in a change in CPQ of almost all CPQ domains of moderate effect size (Table 6). The largest changes (0.5–0.6) were reported in the domains “Functional limitations” and “Oral symptoms”.

**Table 7:** Influence of sociodemographic and malocclusion on CPQ changes (T2): multivariate linear regression analysis

	Estimate	Standard Error	CI95%	*P*- value
**CPQ total**				
Age	-0.06	1.43	-2.86; 2.73	0.966
Sex (Male)	1.31	2.73	-4.05; 6.67	0.633
IOTN AC	0.16	0.63	-1.08; 1.40	0.800
** IOTN DHC 5**	**7.35**	**2.95**	**1.57; 13.13**	0.015*
DMFT equal 0	0.43	2.83	-5.12; 5.98	0.879
**Oral symptoms**				
Age	0.26	0.37	-0.47; 0.98	0.494
Sex (Male)	0.66	0.71	-0.74; 2.06	0.358
IOTN AC	0.02	0.16	-0.30; 0.34	0.903
IOTN DHC 5	0.61	0.77	-0.90; 2.11	0.433
DMFT equal 0	0.10	0.74	-1.35; 1.55	0.891
**Functional limitations**				
Age	-0.71	0.62	-1.93; 0.50	0.254
Sex (Male)	0.43	1.19	-1.89; 2.75	0.717
IOTN AC	-0.21	0.27	-0.74; 0.33	0.450
IOTN DHC 5	1.06	1.82	-1.44; 3.57	0.408
DMFT equal 0	-0.46	1.23	-2.86; 1.95	0.712
**Social well-being**				
Age	-0.04	0.45	-0.92; 0.83	0.925
Sex (Male)	0.49	0.86	-1.19; 2.17	0.567
IOTN AC	0.19	0.20	-0.20; 0.57	0.349
** IOTN DHC 5**	**2.54**	**0.92**	**0.73; 4.35**	0.007*
DMFT equal 0	0.23	0.89	-1.51; 1.97	0.800
**Emotional well-being**				
Age	0.40	0.51	-0.60; 1.39	0.426
Sex (Male)	-0.02	0.97	-1.93; 1.87	0.977
IOTN AC	0.20	0.22	-0.23; 0.64	0.365
** IOTN DHC 5**	**3.21**	**1.04**	**1.17; 5.26**	0.003*
DMFT equal 0	0.49	1.01	-1.48; 2.45	0.627

Note: Regression analysis was conducted for continuous (age, IOTN AC) and dichotomous (sex, IOTN DHC 5, DMFT) variables. Abbreviation: CI, confidence interval. * *P* values considered statistically significant (*P* < 0.05)

After adjustment of the CPQ change by accounting for the effect of confounding variables such as age, sex, IOTN and DMFT-score using linear regression analysis, it was found that a significant relationship (*p* = 0.015) existed between the change in self-reported QoL and IOTN DHC malocclusion during the initial phase of orthodontic treatment (Table 7). More precisely, patients with an IOTN DHC 5 malocclusion exhibited a higher increase in CPQ scoring, amounting to 7.35 points more than patients with an IOTN DHC 4 grade. Notably, this change in CPQ scores was only statistically significant in the domains of Social and Emotional Well-being (*p* = 0.007 and *p* = 0.003, respectively).

## Discussion

The present study was the first study evaluating OHRQoL in Austrian adolescents during the first six months of orthodontic treatment. According to the present results, orthodontic treatment has an initial impact on OHRQoL, but CPQ scores then improve and approach baseline values. However, the domains “social well-being” and “emotional well-being” immediately improved after onset of orthodontic treatment. The impact on OHRQoL was mainly influenced by type of malocclusion.

The development of OHRQoL in adolescents after onset of orthodontic therapy has already been investigated in previous studies [14, 11, 12, 16, 26–28]. Zhang et al. conducted a trial on orthodontic patients with a mean age of 13.1 years in China, who reported on a mean baseline CPQ total score of 20.7 ± 14.4 [16]. Adolescents from Belgium with a mean age of 12.7 years reported on a mean baseline CPQ total score of 17.0 [27]. The children included in our trial were slightly younger, with a mean age of 12.1 years. However, the mean baseline CPQ total score of 17.65 ± 11.06 was not substantially different from the aforementioned studies. In contrast, Abreu et al. conducted a study with Brazilian adolescents with a mean age of 11.5 years who reported a substantially lower baseline CPQ score of 12.10 ± 7.75 points [14].

Despite an observed temporary worsening of overall OHRQoL, two studies reported a decrease in the emotional domain of the CPQ score: Jaeken et al. [27] observed a decrease of 0.9 points after one year of treatment and Zhang et al. [16] a decrease of 1 point after one month. These findings were confirmed in our study with a decrease of 1.82 points after one month and of 1.62 points after six months. However, our patients also experienced a positive effect in the social domain, in contrast to the aforementioned studies. It has been suggested that females are more affected by malocclusion compared to male patients [28, 29], and also experience greater impairment in OHRQoL after start of treatment [26, 30]. In our study, no differences were found between female and male patients.

Only a couple of studies reported on longitudinal data on this topic so far [12, 14, 16, 27] but did not discuss the influence of malocclusion in detail. A recent review confirmed an association of OHRQoL with specific malocclusions in the aesthetic zone, such as anterior crowding, diastema between incisors and increased overjet [8]. Further, it has been indicated that Class I patients benefit most in the stage of alignment, whereas Class II patients reported greatest improvement during space closure after extraction of premolars [12]. In our study, patients with the most severe type of malocclusion (IOTN DHC 5) had the lowest baseline OHRQoL. Accordingly, patients with this malocclusion experienced the least impact of orthodontic treatment on QHRQoL (+ 1.08 CPQ change). The initial IOTN AC grading did not have influence on the development of CPQ during orthodontic treatment.

As recommended in a study assessing methodological quality of measurement of child oral health-related quality of life, this study was supplemented with sociodemographic and clinical data, such as age, sex and malocclusion [20]. All adolescents included in the study presented severe malocclusion, and therefore comprised a homogenous study population. They were all about the same age (11–14 years old), and were treated at the same department accordingly to the requirements provided by the Austrian health service with similar orthodontic appliances (i.e. archwire sequence and bracket system). However, the type of malocclusion (for example overjet, crowding, impacted teeth), except for being classified as IOTN DHC grade 4 or 5, substantially differed among the patients. This variety could not be fully addressed in this study, as too many inhomogeneous groups would have been the result. An attempt has been made to account for the influence of IOTN grading on the change of OHRQoL after start of orthodontic treatment. Treatment was conducted by several clinicians employed at the department; a limitation that might influence outcome data, but has also been accepted by other authors [14, 26].

After initially focusing on adults, questionnaires adapted for children and adolescents have been implemented [31–35]. Currently, several validated instruments exist to assess children’s OHRQoL. Amongst them, the Child Perceptions Questionnaire [31] has been widely tested and validated [20, 36] and has already been used in the orthodontic field [13, 14]. Different language versions exist, making the questionnaire a promising tool for international collaboration [25, 37, 38]. Other validated instruments are the Pediatric Oral Health-Related Quality of Life (POQL) [34], Early Childhood Oral Health Impact Score (ECOHIS) [35], Child Oral Impacts on Daily Performances (Child-OIDP) [33], and the Child Oral Health Impact Profile (COHIP) [32]. The variety of instruments to assess children’s OHRQoL can make it difficulat to compare results between studies. In addition, the use of instruments designed to validate OHRQoL in adults may lead to invalid results when used in children [11].

There is some controversy on the choice of the right malocclusion index to reliably access the impact of occlusal traits on OHRQoL. Different orthodontic indices reveal different associations with CPQ scores amongst patients, as some indices (DAI, IOTN AC) implement aesthetic standards, whereas others (IOTN DHC, ICON) solely rely on occlusal traits [39]. IOTN AC is ought to grade the patients’ frontal aesthetics and is therefore expected to impact social attractiveness, i.e. the social emotional domain of OHRQoL [30, 40]. IOTN DHC focuses on occlusal traits that might not primarily affect OHRQoL, such as impacted teeth or posterior crossbites in an early stage, as suggested in a narrative review [39], whereas Sun et al. detected effects of IOTN DHC on both the functional and the social well-being domain. This study evaluated both IOTN AC and DHC to account for these differences.

As there exists no universal MID (Minimally Important Difference) with threshold values for interpretation of clinical relevance [41], the effect size has been assessed as an approximation of MID. Although statistically significant, the absolute change of OHRQoL reported in this study does not necessarily provide the clinician with information on its clinical relevance or guidelines to change treatment practices. Estimation of effect size comprises the most general approach for MID determination [41], and has been adopted in another study on OHRQoL changes during orthodontic treatment [14]. Still, it only offers an approximation for the MID, as there is no external reference point for interpretation. In our investigation, we assessed patients six months after commencing treatment and observed a moderate effect on the “Oral symptoms” domain. In contrast, Abreu et al. [14] examined patients one year after initiating orthodontic treatment and reported only a small effect. This disparity might be attributed to the initial adjustment phase of patients to the oral appliance.

The multivariate linear regression analysis (Table 7) showed that the DHC score had a significant impact on CPQ changes during orthodontic treatment, suggesting a 7.35-point increase of CPQ in the DHC 5 group compared to the DHC 4 group. However, the wide confidence interval of 1.57–13.13 indicates a significant instability of the estimate. Subgroup analysis of the four CPQ domains showed that the type of malocclusion affected only two CPQ domains: emotional and social well-being, for which the confidence intervals were much smaller. However, the observed instability of the estimate may also be due to the great variation in types of malocclusion, as displayed in Table 1. From the patient’s perspective, a large overjet, that results in an IOTN DHC 4a or 5a grading, may have less impact on OHRQoL when compared to a retained and displaced tooth (IOTN DHC 5i), considering the possibility of a future surgical procedure and uncertain outcome of the aligning of the respective tooth. This underscores the need for further research to explore the impact of malocclusion on changes in OHRQoL during orthodontic treatment in different settings.

This study identified the type of malocclusion as a significant factor affecting adolescents’ quality of life while undergoing orthodontics. However, the small sample sizes within certain malocclusion subgroups may limit the statistical power required to detect meaningful differences or patterns. This challenge arises from the wide variety of malocclusion types present in orthodontic patient populations, highlighting the need for a larger subgroup sample size. Another limitation of the current study was the short follow-up period of the study, which limited the ability to fully explore the longitudinal effects of orthodontic interventions on the daily experience and overall satisfaction of children with malocclusion. Longer observation periods are essential as the impact on quality of life may vary throughout treatment and initial perceptions after treatment may differ significantly from those observed during or at the end of treatment.

The findings of the current study may help to increase knowledge of adolescents’ needs and possible emotional or functional limitations in the initial orthodontic treatment phase. Changes in OHRQoL after onset of treatment might differ substantially among patients. This more profound understanding might help clinicians to individually assess patients benefits and impairments and the resulting responsiveness might further improve patients’ compliance and coping capacities. Future research might not only focus on the first six months but the whole treatment period, including assessment of OHRQoL after orthodontic treatment to provide deeper insights into the lasting effects of orthodontic treatment, and to determine QoL changes with larger sample sizes of malocclusion subgroups. Furthermore, development of a health-related quality-of-life scale with context to clinical relevance would be desirable.

## Conclusion

At the beginning of orthodontic treatment, the OHRQoL slightly worsens, probably due to the discomfort and appearance of the appliances. However, 6 months after the start of orthodontic treatment, OHRQoL improved again in patients with severe malocclusion, and approached baseline values. Patients with an IOTN DHC 5 malocclusion exhibited a higher increase in CPQ scoring. The largest changes were observed in the domains “Functional limitations” and “Oral symptoms”.
